# Martini 3 Limitations in Phospholipid Flip-Flop

**DOI:** 10.1021/acs.jctc.5c00994

**Published:** 2025-09-25

**Authors:** Ondřej Kroutil, Ladislav Bartoš, Ivo Kabelka, Robert Vácha

**Affiliations:** † CEITEC−Central European Institute of Technology, 37748 Masaryk University, 625 00 Brno, Czech Republic; ‡ National Centre for Biomolecular Research, Faculty of Science, Masaryk University, 625 00 Brno, Czech Republic

## Abstract

Phospholipid membranes serve as essential barriers in biological systems, and protein-mediated lipid flip-flop is a crucial process for lipid homeostasis in membranes, which is vital for various cellular functions. These phenomena can be studied using the Martini coarse-grained force field, a valuable tool for membrane simulations that balances computational efficiency with chemical accuracy while capturing key membrane properties. However, the accuracy of the newer Martini 3 force field in describing energetics of phospholipid flip-flop remains unknown. Here, we show dramatic differences in the free energy barriers of lipid flip-flop when simulated with Martini 3, Martini 2.2, and CHARMM36m force fields. Using umbrella sampling simulations of six phospholipids (POPC, DPPA, POPE, POPG, POPS, and DPTAP) in the POPC membrane, we demonstrate that Martini 3 predicts significantly lower flip-flop barriers compared to all-atom simulations and the older Martini 2.2 version, with particularly severe underestimation for the positively charged lipid (DPTAP). For DPTAP, we identified that altered Lennard-Jones parameters between choline and alkyl tail beads likely contribute to this discrepancy, as evidenced by our systematic parameter testing. These findings highlight limitations in the current Martini 3 parametrization that should be considered when studying processes involving molecular transport across membranes and suggest potential refinements to improve the model’s accuracy for such phenomena. To complete the picture, we also discuss the energetics of flip-flops of phospholipids with various lipid tail lengths (DLPC, DMPC, DPPC, DSPC) and lipid tail saturation (DOPC).

## Graphical abstract

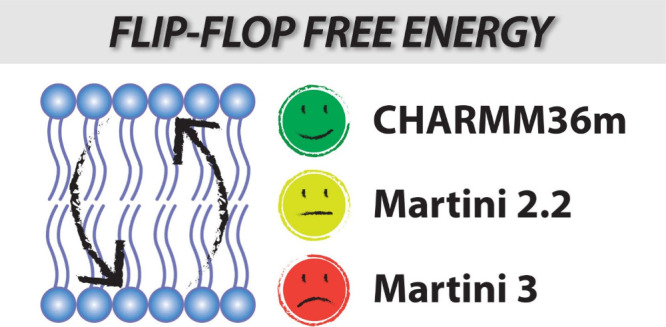

Transport of molecules across the semipermeable lipid membrane is a biologically vital process. Lipids themselves can undergo transbilayer movement through a process known as flip-flop, which is critical for maintaining membrane asymmetry and homeostasis. It is an energetically demanding process, as it involves the passage of hydrophilic lipid headgroup through the membrane’s hydrophobic core. For common phospholipids, experimental and theoretical studies have reported free energy barriers for flip-flop ranging from 80 to 220 kJ mol^–1^, with half-times on the order of minutes to hours.^1^ These values depend strongly on lipid type, membrane composition, and environmental conditions.^2^

Coarse-grained force fields reduce computational complexity by representing multiple atoms as single interaction sites (beads), enabling simulation of larger systems over extended time scales while sacrificing chemical specificity and fine-grained interactions. The Martini force field, with its 4:1 mapping strategy (on average), has become particularly valuable for membrane simulations by effectively balancing efficiency and chemical accuracy. Martini successfully reproduces key membrane properties including area per lipid, bilayer thickness, and lipid diffusion rates while capturing phase transitions and domain formation in multicomponent systems.^3^ Its parametrization based on free energies of transfer between polar and apolar environments makes it especially suitable for membrane-associated processes.

Since its release in 2021,^4^ Martini 3 has been applied to a variety of systems, prompting several modifications aimed at improving its accuracy for particular cases. For example, studies on protein–water interactions have reported conflicting results, with the nature of the issues varying depending on the system. For intrinsically disordered proteins and multidomain proteins in solution, Martini 3 produces overly compact structures, suggesting that protein–water interactions are underestimated^5,6^ while for transmembrane proteins and peripheral proteins, Martini 3 often fails to capture membrane insertion, suggesting protein–water interactions are overestimated.^7,8^ A study by Spinti et al.^9^ reported that WALP peptides (particularly shorter ones like WALP16) tend to inappropriately exit the membrane and adopt surface-adsorbed states. From a statistical mechanics perspective, Jarin et al.^10^ and later Loose et al.^11^ have concluded that both Martini 2.2 and Martini 3 coarse-grained force fields have significant drawbacks when compared to all-atom models. Despite improvements, Martini 3 still fails to accurately decompose free energy into enthalpic and entropic components, compared to mapped atomistic simulations. Martini 3 also fails to capture the temperature-dependent behavior of saturated lipid tails, such as those in DPPC, largely due to missing entropic contributions.^10,11^ Additionally, the model continues to struggle with accurately representing interactions between different headgroups, particularly for charged lipids like DOPS.

In this study, we report another limitation of the Martini 3 force field, namely the inconsistent predictions of flip-flop free energy of neutral, anionic, and cationic phospholipids.

## Comparison of the Calculated Flip-Flop Barrier Values

Using umbrella sampling (US) simulations, we calculated flip-flop free energy profiles for selected six phospholipids with different formal charges (POPC/0*e*, POPE/0*e*, DPPA/-1*e*, POPG/-1*e*, POPS/-1*e*, and DPTAP/+1*e*) in a POPC membrane, employing four force fields, namely CHARMM36m,^12^ Martini 2.2,^13^ Martini 3^4^ and refined Martini 3 parameters published in^14^ and currently available as version 2 (referred to as “Martini 3_v2” hereafter). To complete the picture, we simulated also lipids with various tail lengths (DLPC/12:0, DMPC/14:0, DPPC/16:0, and DSPC/18:0) and saturation (DOPC/18:1).

First, we evaluated the free energy barriers of flip-flop. In all-atom simulations, the height of the flip-flop free energy barrier (ΔG_F–F_) was following for six lipids with different headgroups - POPC (92 kJ mol^–1^), POPE (89 kJ mol^–1^), DPPA (89 kJ mol^–1^), POPG (86 kJ mol^–1^), POPS (104 kJ mol^–1^) and DPTAP (84 kJ mol^–1^). Except for the POPS molecule, which is discussed below, the final flip-flop barriers indicate minimal dependence on the headgroup charge with an estimated error of 6 kJ mol^–1^ (see Figure 1 and Table S1).

**Figure 1:**
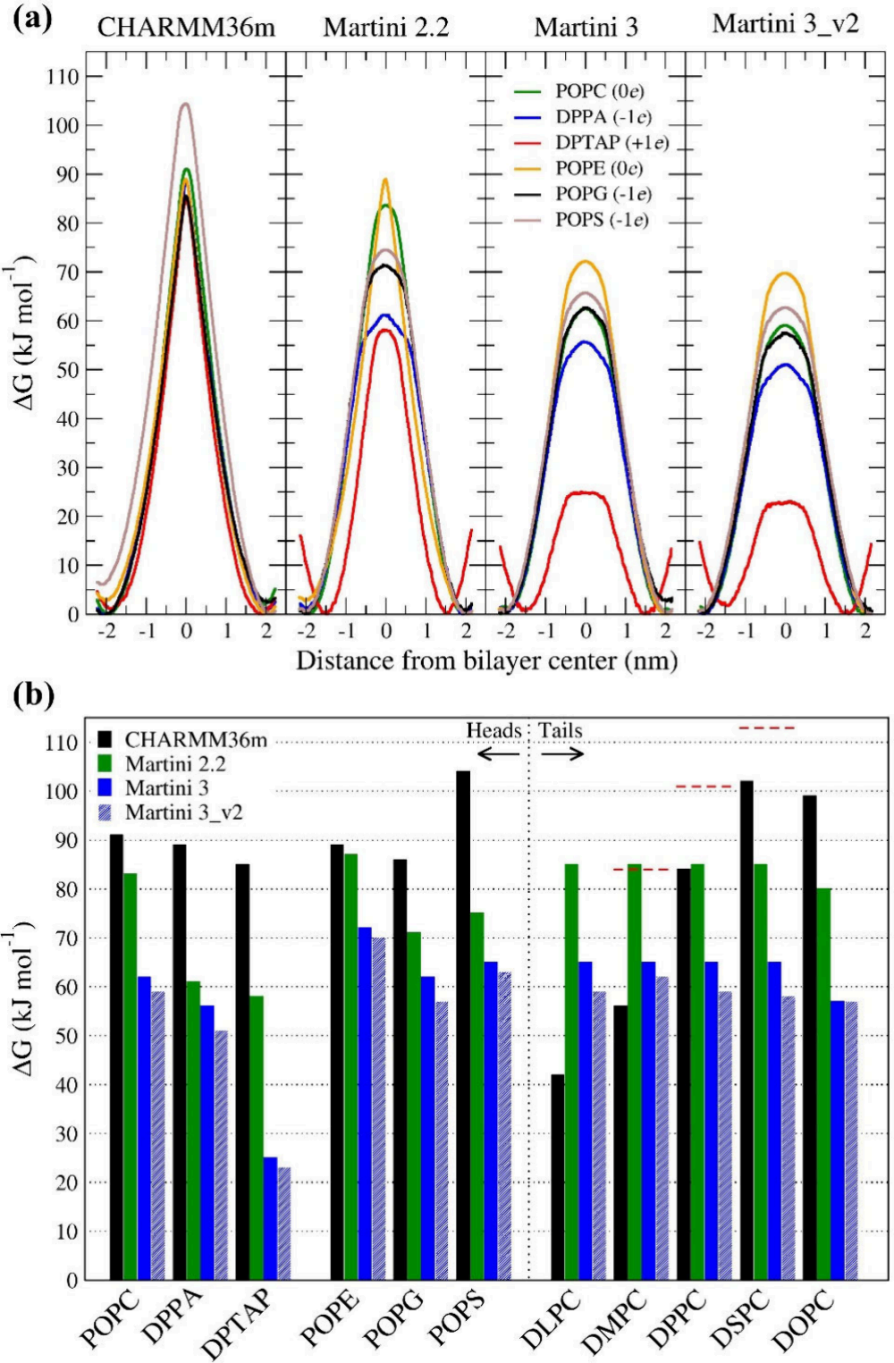
(a) Free energy of flip-flop for six phospholipids with different head groups (zwitterionic POPC, POPE; negatively charged DPPA, POPG, and POPS; and positively charged DTAP in POPC membrane) in CHARMM36, Martini 2.2, Martini 3, and Martini 3_v2 force fields. From asymmetry, we estimate the error of the profiles to be 6 and 3 kJ mol^–1^ for CHARMM36m and Martini profiles, respectively. (b) Bar chart of the free energy barriers of the flip-flop for all 11 studied phospholipids. Red horizontal dashed bars over DMPC, DPPC, and DSPC show experimental values taken from Anglin et al.^1^

In contrast, CG simulations using the Martini force field displayed a strong deviation from these values. With Martini 2.2, the ΔG_F–F_ for POPC was 83 kJ mol^–1^ (∼10% lower than the AA value) with estimated error 3 kJ mol^–1^, which could be related to the simplified representation and the lack of key interactions such as hydrogen bonding. Martini 3 showed an even more substantial decrease in ΔG_F–F_ for zwitterionic POPC (63 kJ mol^–1^, 68% of the AA value), suggesting deeper parametrization issues. The underestimation was more pronounced for charged lipids: in Martini 2.2, ΔG_F–F_ values for DPPA and DPTAP were reduced by ∼30% (61 and 58 kJ mol^–1^, respectively), while in Martini 3, the reductions were even greater - DPPA dropped by ∼38% (56 kJ mol^–1^), and DPTAP by ∼70% (25 kJ mol^–1^). Calculated values from Martini 3_v2 were ∼1% lower still, highlighting further divergence. The decrease in flip-flop energy for neutral POPE was ∼2% and ∼20% with Martini 2.2 and Martini 3, respectively, compared to all-atom. For negatively charged POPG and POPS, the decrease was 20–30% and 30–40% for Martini 2.2 and Martini 3.

These simulation results were compared with previously published experimental and computational studies. Experimental studies also found only minor differences in the activation energy of flip-flop between zwitterionic and negatively charged lipids. For instance, a study on DPPS (−1*e*) in a DSPC (0*e*) membrane^15^ reported an activation energy (barrier) of the DPPS flip-flop ∼10 kJ mol^–1^ lower than that of DSPC. Another experiment showed DSPG’s activation energy to be ∼2 kJ mol^–1^ lower than DSPC in a single-component bilayer.^16^ Earlier research using pyrene-labeled lipids^17^ indicated that the free energy for flip-flop was approximately 4 kJ mol^–1^ lower for negatively charged headgroups (PG and PA) than for choline-containing lipids. However, computational studies using different force fields sometimes showed the opposite trend. We found an energy barrier of 104 kJ mol^–1^ for the POPS flip-flop in the POPC membrane, which is 12 kJ mol^–1^ higher than for the neutral POPC. Using the united-atom Berger force field, a greater barrier of 120 kJ mol^–1^ was found for the same system.^18^ Conversely, Razzokov et al.^19^ obtained a flip-flop barrier of 93 kJ mol^–1^ with the united-atom GROMOS 43A1-S3 force field, which is close to the POPC value. Using unbiased molecular dynamics and artificial intelligence, Post and Hummer recently discovered^20^ that lipid flip-flop can occur through different mechanisms (tunneling, nanopore formation, or transient water threads) depending on the thickness of the membrane and the type of lipid. Differences in the aforementioned barriers thus could arise also from the inadequate sampling of the proper mechanism in different force fields and/or the simulation method. Regarding the other charged phospholipid, POPG, simulations of DOPG in a DOPC membrane using all-atom OPLS-AA^21^ and united-atom Berger’s force field^22^ yielded a ΔG_F–F_ value 18 kJ mol^–1^ higher than for DOPC. To our knowledge, no previous experimental or computational study has determined the activation energy for positively charged phospholipids.

As described above, we observed a slightly lower free energy barrier (∼5 kJ mol^–1^) for positively charged lipids compared to negatively charged ones. Given the small magnitude of this difference, our findings support the conclusion that the flip-flop free energy barrier is rather independent of the headgroup charge. This conclusion is consistent with observations from studies on charged amino acids, where the free energies of translocation for positive and negative residues were also found to be very similar.^23,24^ Although some discrepancies exist between individual studies, the overall trend supports our conclusion that the lipid flip-flop barrier is minimally affected by headgroup charge.

It is a known fact that electrostatic interactions are poorly captured in the Martini 2.2.^23^ The problem under consideration is directly related to the nature of the Martini force field and the way it defines the water bead. The standard Martini water model represents four water molecules as a single, uncharged bead, devoid of any dipole moment. This model exhibits behavior more akin to a vacuum in terms of electrostatic screening. Consequently, the electrostatic potential in the membrane does not exhibit slight positive values, as observed in all-atom simulations^24^ and experiments,^25,26^ but rather, it approaches negative values within the membrane (see Figure S1). On the other hand, the parametrization of the Martini 2 force field was able to compensate for these shortcomings by a choice of LJ parameters.

To further explore the differences between force fields in the description of flip-flop energetics, we focused on PC phospholipids with lipid tails of varying lengths and saturations (Figure 1(b), Figure S2 and Table S1). In our investigation of the influence of lipid chain length on the free energy of flip-flop, we discovered that none of the examined coarse-grained force fields could reproduce the experimentally measured trend. Experiments have shown that the longer the lipid chain, the greater the energy required for phospholipid flip-flop.^1^ While all-atom simulations qualitatively follow this trend, the absolute values of ΔG_F–F_ are ∼30–10 kJ mol^–1^ lower than in the experiment. Conversely, neither Martini 2.2 nor Martini 3 can reproduce this trend. These force fields have a lower resolution; therefore, DLPC and DMPC pairs, as well as DPPC and DSPC pairs, are always represented by one molecule with three or four beads in the lipid tail, respectively. Therefore, there is no difference in the flip-flop barrier between DLPC/DMPC and DPPC/DSPC either in Martini 2.2 or in Martini 3. The absolute values in Martini 3 are about 25% lower than those in Martini 2.2, just like in the POPC case. The Martini 3_v2 force field introduces the SC1 bead, which has a smaller radius than the C1 bead. This change allows it to distinguish between lipid tails that differ by two carbons. However, even in this case, all ΔG_F–F_ values were essentially the same for all PC phospholipids.

The above observations show that all Martini force fields poorly capture the energetics of phospholipid flip-flops in the membrane. At the same time, it is clear that Martini 3 is much worse than Martini 2.2, and the newly reparameterized lipids in the Martini 3_v2 force field performs even worse in describing flip-flop energetics.

## Possible Origins of the Martini 3 Failure

A comparison of all Lennard-Jones parameters for POPC shows that the authors mainly modified the epsilon values except for the Q1–C1 case discussed in the paper. Most of the epsilon values for the choline and phosphate beads were increased including those with lipid tails, which resulted in a more flexible membrane but also an easier lipid flip-flop across the membrane.

Since any force field is a delicate tangle of parameters, each related to the others, it is difficult to point to a single parameter that could account for such different flip-flop results between the Martini 3 (and Martini 3_v2) and other force fields. A closer look at all the Lennard-Jones (LJ) parameters of the lipid bead combinations shows a striking difference, which is the sigma value of the interaction between the choline bead Q1 and the C1 bead of the lipid tails (see Table S2). While in most other LJ parameters only the epsilon value changed significantly between Martini 2.2 and Martini 3 force fields, the sigma value decreased from 0.620 to 0.485 nm, i.e. by a full 0.135 nm, for the interaction between beads Q1 and C1 (see Figure 2(a) for definition). The changes indicate that the membrane interior is less hydrophobic for the choline bead, which can therefore pass through it more easily. In the case of the interaction of the phosphate bead with C1 beads, there was only a slight increase in the epsilon in the LJ interaction compared to the values in Martini 2.2. This means the same hydrophobicity of the center of the membrane for the phosphate bead. We speculate that this difference in the LJ parameters could be one of the reasons why DPTAP behaves so differently in Martini 3 and Martini 2.2.

**Figure 2:**
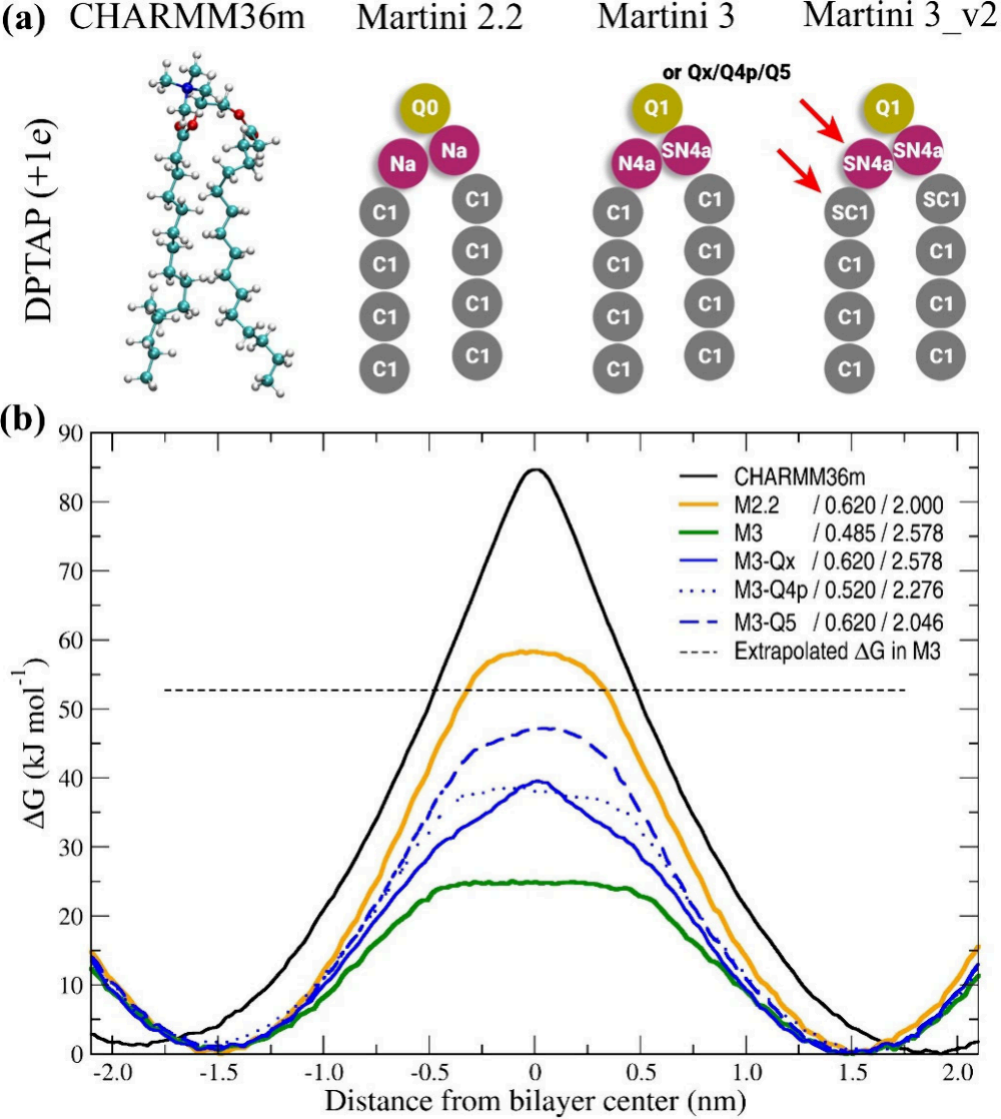
(a) Model of the DPTAP in different force fields. For Martini force fields, the atomic types are indicated, and differences between Martini 3 and Martini 3_v2 are highlighted with a red arrow. The differences are discussed in more detail in the SI (Table S2). (b) Free energy flip-flop of the DPTAP with different force fields and different Lennard-Jones parameters for Q1–C1 interactions. The values of the sigma (nm) and epsilon (kJ mol^–1^) are given in the legend. We calculated the extrapolated ΔG value for DPTAP in Martini 3 from the same ΔG ratio between DPPA and DPTAP as is found in Martini 2.2.

To provide support for this idea, we performed simulations in the DPTAP flip-flop using modified Martini 3 force field with varying the magnitude of the sigma and epsilon parameters of the LJ interaction between Q1 and C1 beads (Figure 2(b)). The tested values of the sigma and epsilon are summarized in the Table 1. For the other LJ parameters, the original choline bead sigma and epsilon values were retained.

**Table 1:**
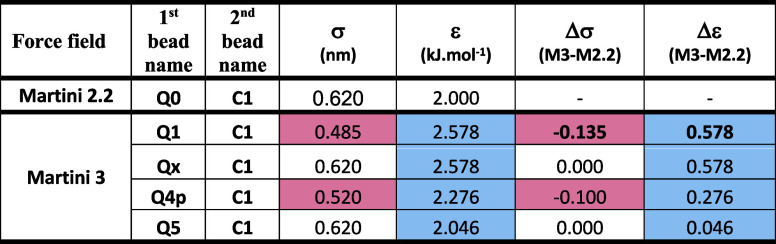
Lennard-Jones Parameters for Interactions of Choline Bead and C1 Tail Bead^a^

a Red color means decrease in value compared to the Martini 2.2 reference value. Blue color means increase in value. Qx is an artificial bead with the same sigma value as in M2.2. Q4p is an ethanolamine bead, and Q5 is a phosphate bead.

As discussed earlier, new Martini 3 LJ values result in 57% lower flip-flop free energy barrier compared to Martini 2.2 values. Assuming the same ratio between DPTAP and DPPA flip-flop barriers in Martini 2.2 (95%), the extrapolated ΔG of DPTAP in Martini 3 should be 53 kJ mol^–1^ (Table S1). This extrapolated value is about twice as big as in Martini 3. Since the main difference between two Martini force fields is the value of sigma, we first tested assigning the same value of sigma to the choline bead in Martini 3 as in Martini 2.2 (bead Qx; σ = 0.620 nm), while keeping the actual value of epsilon unchanged (ε = 2.578 kJ mol^–1^). This change resulted in an increase of the free energy barrier to 39.5 kJ mol^–1^ (75% of extrapolated value) but slightly deformed the shape of the free energy curve. Then we tested Martini 3 LJ parameters for ethanolamine bead (Q4p) with sigma and epsilon parameters, which are falling approximately between Martini 2.2 and Martini 3 values for choline beads (see Table 1). These parameters resulted in a slight decrease in flip-flop ΔG (73% of extrapolated value) and the shape of the free energy curve more closely follows the curve from the Martini 2.2 simulations. Finally, we tested for choline bead LJ values of the phosphate bead Q5 that are in Martini 3 very close to original Martini 2.2 values for Q0-C1 interactions. With this setting, the flip-flop ΔG value jumped to 47 kJ mol^–1^, i.e. 89% of the extrapolated ΔG for DPTAP flip-flop. Based on these findings, we hypothesize that altered interactions of choline beads with C1 beads and thus reduced hydrophobicity of the membrane interior likely underlies the reduced flip-flop barrier in zwitterionic POPC phospholipids.

Although the Martini 3 force field has improved predictions for properties such as gel–liquid transition temperatures and phase separation in ternary mixtures,^14^ this letter demonstrates that its performance in predicting flip-flop energetics is worse than that of the previous parametrization when compared to all-atom simulations or experimental data. In addition, our results suggest that Lennard-Jones parameters between the choline bead and the C1 bead of the phospholipid tails might be the origin of this altered barrier. Our results suggest that the current Martini force field parametrization should be used with caution when studying molecular transport across lipid membranes, despite its widespread adoption for this very purpose. For instance, Martini 3 is frequently applied to study the activity of scramblases,^27–31^ proteins that facilitate energy-independent lipid flip-flop. In fact, the popularity of Martini 3 for this purpose likely stems from its reduced flip-flop barriers, which enable scrambling to be observed in unbiased molecular dynamics simulations at achievable time scales. However, our findings raise concerns about the quantitative reliability of such simulations, despite their qualitative agreement with in vitro experimental data.

## Methods

The free energy of lipid flip-flop was calculated for 11 selected phospholipids using program package GROMACS v2021.4. Six selected phospholipids represent different charge states: zwitterionic 1-palmitoyl-2-oleoyl-sn-glycero-3-phosphocholine (POPC; charge 0*e*), 1-palmitoyl-2-oleoyl-sn-glycero-3-phosphoethanolamine (POPE; charge 0*e*); negatively charged 1,2-dipalmitoyl-sn-glycero-3-phosphatidic acid (DPPA; charge −1*e*), 1-palmitoyl-2-oleoyl-sn-glycero-3-phosphoglycerol (POPG; charge −1*e*), 1-palmitoyl-2-oleoyl-sn-glycero-3-phospho-l-serine (POPS; charge −1*e*); and positively charged 1,2-dipalmitoyl-sn-glycero-3-trimethylammonium propane (DPTAP; charge +1*e*). Other five phospholipids represent different lipid tail lengths and saturation (1,2-dilauroyl-sn-glycero-3-phosphocholine/DLPC; 1,2-dimyristoy l-sn-glycero-3-phosphocholine/DMPC; 1,2-dipalmitoyl-sn-glycero-3-phosphocholine/DPPC; 1,2-disteraoyl-sn-glycero-3-phosphocholine/DSPC; and 1,2-dioleoyl-sn-glycero-3-phosphocholine/DOPC). The following sections describe the system and simulation setup for both coarse-grained and all-atom simulations.

### System and Simulation Setup for Coarse-Grained (CG) Systems

We used both the older Martini 2.2^13^ and the newer Martini 3^4^ force field. For Martini 3, we evaluated both the original lipid parametrization and the recently published refined version (v2)^14^ of the parametrization. The system and simulation setup were the same for all force fields. The membrane generated by CHARMM-GUI web service^32^ was composed of 255 POPC molecules, with one additional POPC/DPPA/DPTAP/POPE/POPG/POPS molecule. In the case of the flip-flop in the pure membrane, the membrane was composed of 256 DLPC/DMPC/DPPC/DSPC or DOPC phospholipids. Approximately 4900 water beads were added into the system together with ions (Na^+^ and Cl^–^) at ionic strength of 0.15 M and an extra ion for system electroneutrality (if needed). For initial minimization and equilibration phases we have used CHARMM-GUI’s seven-step procedure, which involves decreasing positional and dihedral restraints on phospholipid molecules and increasing the time step. Following the initial stage, a pulling simulation was conducted in which the flip-flopping phospholipid was translocated across the membrane at a rate of 0.0000042 nm·ps^–1^ over a duration of 1 μs, enabling it to traverse from one side of the membrane interface to the other. From this pull trajectory, initial structures for 46 US windows were generated with spacing between windows and force constants used for umbrella sampling summarized in Table S3 in SI. For structures close to membrane center, we used finer parameters (spacing 0.055 nm and a force constant 5000 kJ mol^–1^·nm^–2^) compared to windows close to membrane/water interface (spacing 0.11 nm and a force constant 3000 kJ mol^–1^·nm^–2^). Other simulation parameters were as follows: a leapfrog integrator was used with a time step of 20 fs, a Verlet cutoff scheme was used for neighbor searching with updates every 20 steps, and periodic boundary conditions were applied in all three dimensions. Electrostatic interactions were treated using a reaction-field approach with a cutoff distance of 1.1 nm, a dielectric constant of 15, and a reaction field dielectric of infinity. The van der Waals interactions were also treated with a cutoff at 1.1 nm using the Potential-shift-Verlet modifier. Temperature was maintained at 310 K using the stochastic velocity-rescaling thermostat (V-rescale) with a coupling constant of 1.0 ps, applied separately on membrane and solvent. Pressure was controlled semiisotropically at 1 bar using the Parrinello–Rahman barostat with a coupling time of 12.0 ps and a compressibility of 3 × 10^–4^ bar^–1^.

### System and Simulation Setup for All-Atom (AA) Systems

We calculated the free energy of flip-flop also in the all-atom resolution using the commonly used CHARMM36m force field.^12^ The size of the simulated systems was reduced compared to Martini simulations in order to save computing time. Each system contained 128 phospholipids: 127 POPC + 1 that flip-flops, which may be POPC, DPPA, PPTAP, POPE, POPG and POPS. In the case of the flip-flop in the pure membrane, the membrane consisted of 128 DLPC/DMPC/DPPC/DSPC or DOPC phospholipids, and one phospholipid molecule underwent flip-flop movement. Approximately 5500 water molecules were added to solvate the system, and the number of ions (Na^+^ and Cl^–^) was adjusted to a concentration of 0.15 M, with an additional ion added to maintain electroneutrality. Although the number of phospholipids in the all-atom system is half that of the Martini simulation, the results should not be affected. As was previously demonstrated,^21,33^ even smaller systems containing a total of 64 phospholipids were large enough to provide well-converged potentials of mean force for lipid flip-flop calculations.

Due to slower convergence of AA systems, we used two distinct initial configurations for the purpose of pulling: one with a flip-flopped molecule in the upper leaflet and the other with the molecule in the lower leaflet. These initial configurations were generated by CHARMM-GUI web service^32^ and were equilibrated according to CHARMM-GUI’s seven-step procedure, which involves decreasing positional and dihedral restraints on phospholipid molecules and increasing the time step. Subsequent to this, the process of pulling the flip-flopped molecule to an opposite leaflet was performed, i.e., up–down and down–up pulling. The pulling simulations were executed for a duration of 25 ns, with the pulling rate at 0.000168 nm·ps^–1^. The constant force was set to 8000 kJ mol^–1^·nm^–2^. The phosphate atom or the nitrogen atom (DPTAP) was designated as the pulling group.

Following the pulling phase, the configurations for umbrella sampling were generated with spacing reported in Table S3 in SI. To improve the convergence of the all-atom simulations, we chose Hamiltonian Replica Exchange Molecular Dynamics (H-REMD) for 16 windows covering configurations in the center of the membrane (Table S3). The H-REMD windows used a finer spacing (0.055 nm) with a higher force constant (3000 kJ mol^–1^·nm^–2^) compared to the normal non-REMD windows (spacing 0.11 nm and force constant 2000 kJ mol^–1^·nm^–2^). Exchange between replicas was attempted every 100,000 steps. A 600 ns trajectory was simulated in each window.

The production setup was as follows: time step set to 2 fs; NPT ensemble at 310 K and 1 bar, with temperature controlled by a velocity-rescaling thermostat (τ = 0.5 ps) and pressure controlled by a Parrinello–Rahman barostat (semi-isotropic, compressibility = 4.5 × 10^–5^ bar^–1^, τ = 2.0 ps); long-range electrostatics handled by the Particle-Mesh-Ewald (PME) method with a Fourier spacing of 0.12 nm and a cutoff of 1.2 nm for both electrostatic and van der Waals interactions with the radius switch of 1.0 nm using the force-switch modifier; bond constraints were imposed by the LINCS algorithm on H-bonds.

The umbrella sampling data were analyzed using the weighted histogram analysis method (WHAM)^34^ to construct a potential of mean force (PMF) profile, which provided detailed insights into the free energy barrier associated with phospholipid flip-flop across the membrane. The errors in the PMF data were estimated using the bootstrap algorithm implemented in the gmx wham tool of GROMACS. The resulting errors were 0.5 kJ mol^–1^ for all-atom simulations and 0.3 kJ mol^–1^ for coarse-grained simulations. Additionally, based on the asymmetry of the CHARMM36m profiles, we estimate an uncertainty of approximately 6 kJ mol^–1^ for this specific profile; Martini profiles were, on average, asymmetric by 3 kJ mol^–1^.

## Supplementary Material



## Notes

Electrostatic potential in the POPC membrane, flip-flop’s free energy profiles for DLPC/DMPC/DPPC/DSPC/DOPC, and tables with Lennard-Jones parameters and umbrella sampling settings (PDF)
