# Correction to “Martini 3 Limitations in Phospholipid Flip-Flop”

**DOI:** 10.1021/acs.jctc.6c00296

**Published:** 2026-03-18

**Authors:** Ondřej Kroutil, Ladislav Bartoš, Ivo Kabelka, Robert Vácha

We recently found an error in the free energy flip-flop profile of the DPTAP, where the wrong atom was pulled. Here we provide Figures 1 and 2 with corrected profiles as well as an updated Supporting Information file. The most significant change is for the M3-Q4p and M3-Qx systems, where the change of the barrier height (7 kJ mol^–1^) is beyond the estimated error of the profiles (3 kJ mol^–1^). None of the main discussions and conclusions of the original article are affected, but we would like to replace two sentences.

**Figure 1:**
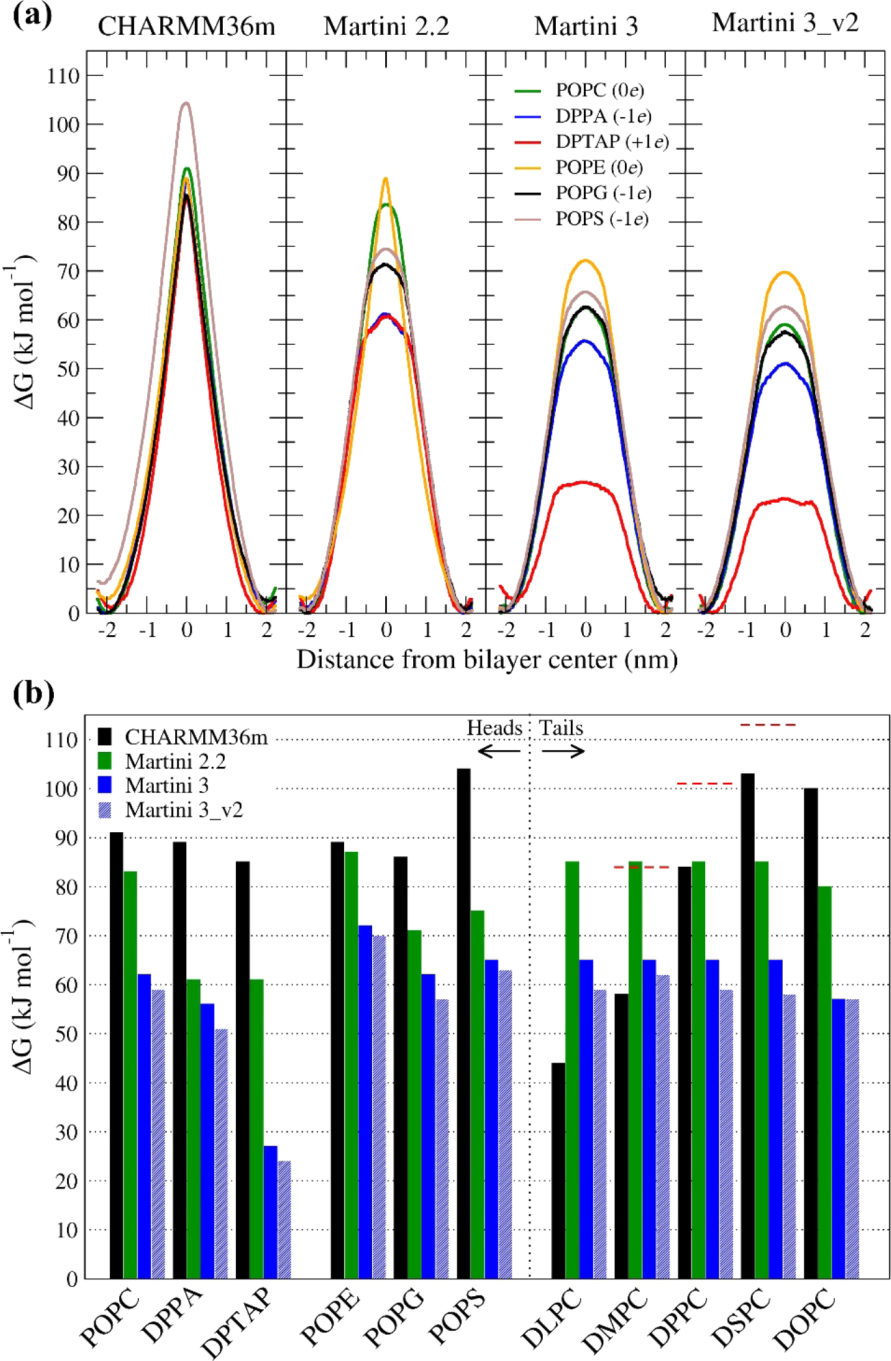
(a) Free energy flip-flop for six phospholipids with different head groups (zwitterionic POPC, POPE; negatively charged DPPA, POPG and POPS; and positively charged DTAP in POPC membrane) in CHARMM36, Martini 2.2, Martini 3 and Martini 3_v2 force fields. From asymmetry, we estimate the error of the profiles to be 6 and 3 kJ mol^–1^ for CHARMM36m and Martini profiles, respectively. (b) Bar chart of the free energy barriers of the flip-flop for all 11 studied phospholipids. Red horizontal dashed bars over DMPC, DPPC and DSPC show experimental values taken from Anglin et al.^1^

**Figure 2:**
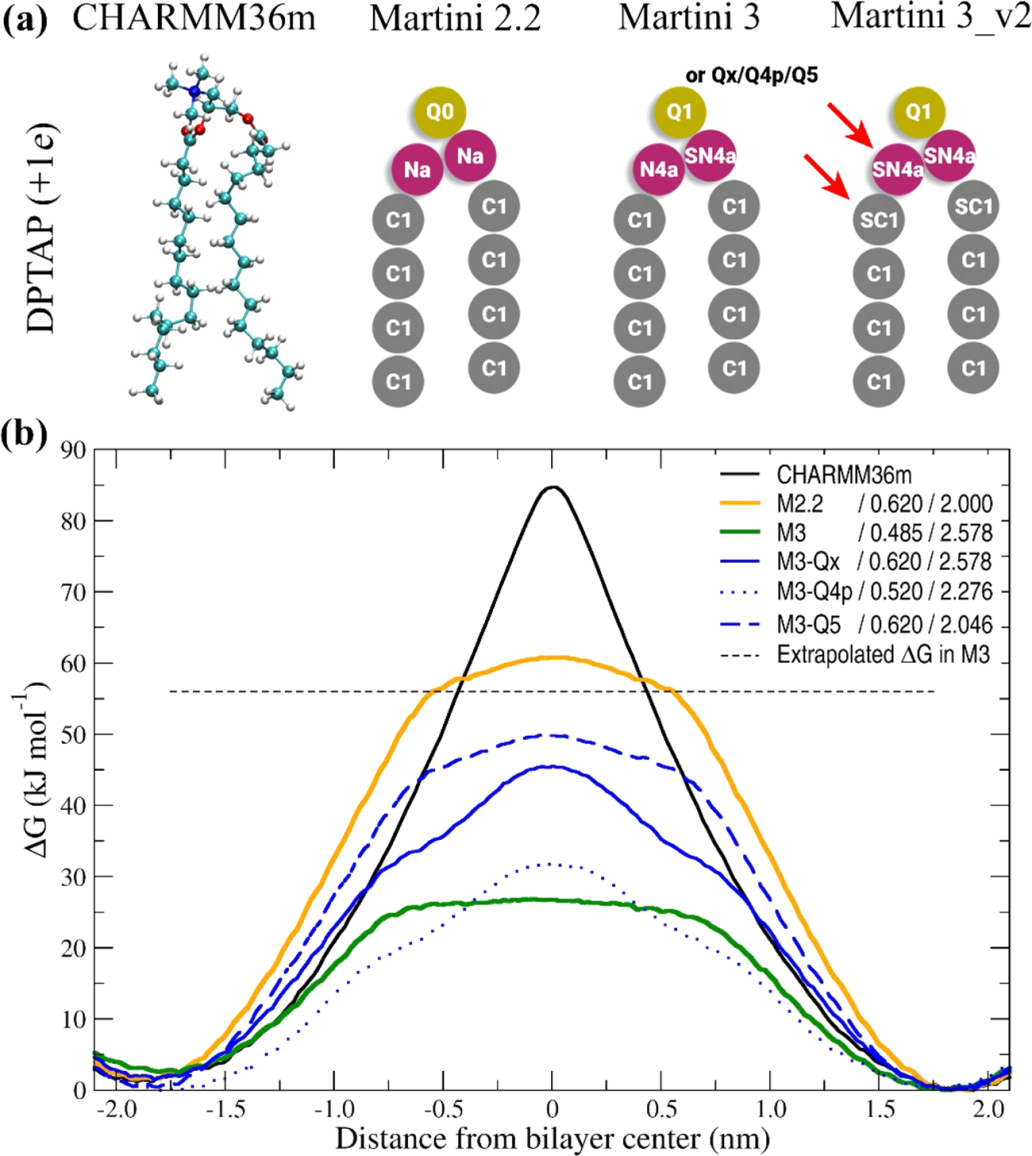
(a) Model of the DPTAP in different force fields. For Martini force fields, the atomic types are indicated and differences between Martini 3 and Martini 3_v2 are highlighted with a red arrow. The differences are discussed in more detail in the SI (Table S2). (b) Free energy flip-flop of the DPTAP with different force fields and different Lennard-Jones parameters for Q1–C1 interactions. The values of the sigma (nm) and epsilon (kJ mol^–1^) are given in the legend. We calculated the extrapolated ΔG value for DPTAP in Martini 3 from the same ΔG ratio between DPPA and DPTAP as is found in Martini 2.2.

Changes in text are as follows:

*Original:*

This change resulted in an increase of the free energy barrier to 39.5 kJ mol^–1^ (75% of extrapolated value) but slightly deformed the shape of the free energy curve.

*Corrected:*

This change resulted in an increase of the free energy barrier to 45.7 kJ mol^–1^ (82% of extrapolated value) but slightly deformed the shape of the free energy curve.

*Original:*

These parameters resulted in a slight decrease in flip-flop ΔG (73% of extrapolated value) and the shape of the free energy curve more closely follows the curve from the Martini 2.2 simulations.

*Corrected:*

These parameters resulted in a notable decrease in flip-flop ΔG (56% of extrapolated value) and the shape of the free energy curve follows the M3-Qx curve.

## Supplementary Material



## Notes

Revised file containing updated values in Table S1 (PDF)

