# Supplementary material for: Martini 3 Limitations in Phospholipid Flip-Flop
Source: J Chem Theory Comput. 2025 Sep 25;21(19):9227–33. doi: 10.1021/acs.jctc.5c00994 (PMC12529914; doi:10.1021/acs.jctc.5c00994)
Supplement: Supplementary file 1 [file ct5c00994_si_001.pdf]

Martini 3 Limitations in Phospholipid Flip-Flop

Ondřej Kroutil[1], Ladislav Bartoš[1,2], Ivo Kabelka[1], Robert Vácha[1,2,*]

[1]CEITEC−Central European Institute of Technology, Masaryk University, 625 00 Brno, Czech Republic;

[2]National Centre for Biomolecular Research, Faculty of Science, Masaryk University, 625 00 Brno, Czech Republic;

[*]Corresponding author: E-mail: robert.vacha@muni.cz, Tel.: +420 549 496 846

Table S1. Free energies of the flip-flop (in kJ.mol^{-1}). Error from bootstrap algorithm for all-atom and coarse-grained simulations was 0.5 kJ.mol^{-1} and 0.3 kJ.mol^{-1}, respectively. From asymmetry of the free energy profile, we estimate the error of the profiles to be 1.5 kJ.mol^{-1} for CHARGM36m and 0.7 kJ.mol^{-1} for Martini.

**Phosholipid**	**Force field**			
	**CHARMM36m**	**Martini 2.2**	**Martini 3**	**Martini 3_v2**
POPC (0*e*)	92	83	63	59
POPE (0*e*)	89	87	72	70
DPPA (-1*e*)	89	61	56	51
POPG (-1*e*)	86	71	62	57
POPS (-1*e*)	104	75	65	63
DPTAP (+1*e*)	84	58	25	23
DLPC	42	85	65	59
DMPC	56	-	-	62
DPPC	84	85	65	59
DSPC	102	-	-	58
DOPC	99	80	57	57

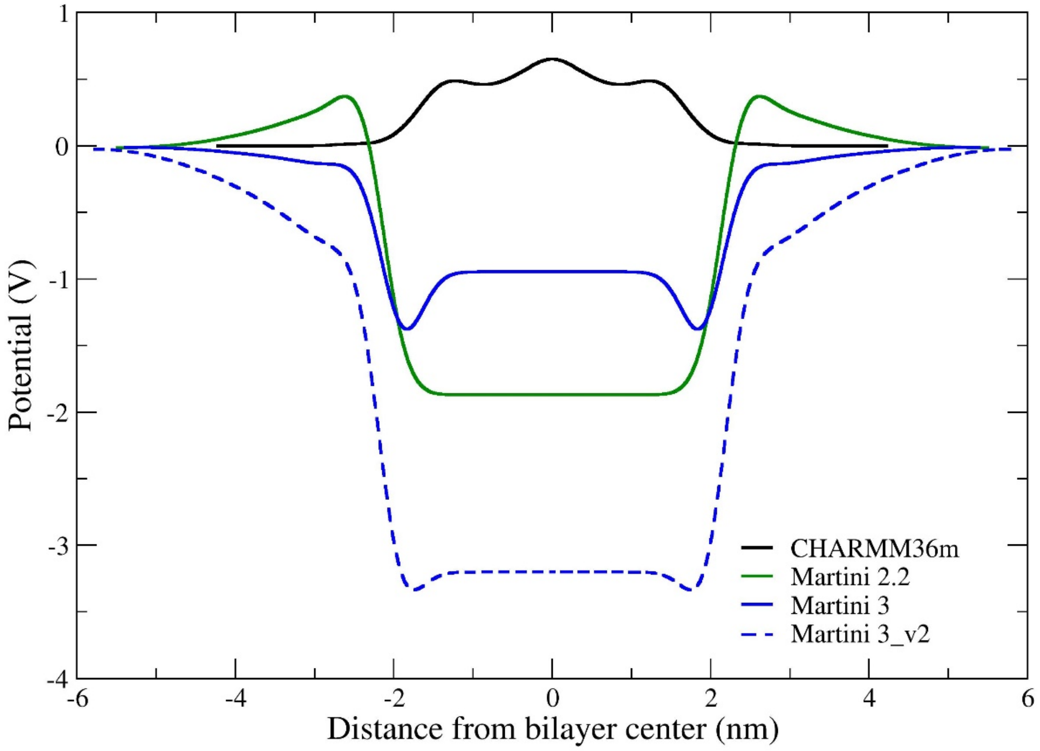

Figure S1. Electrostatic potential in the POPC membrane computed for all studied force fields. Profiles were symmetrized with respect to the center of the membrane (z = 0).

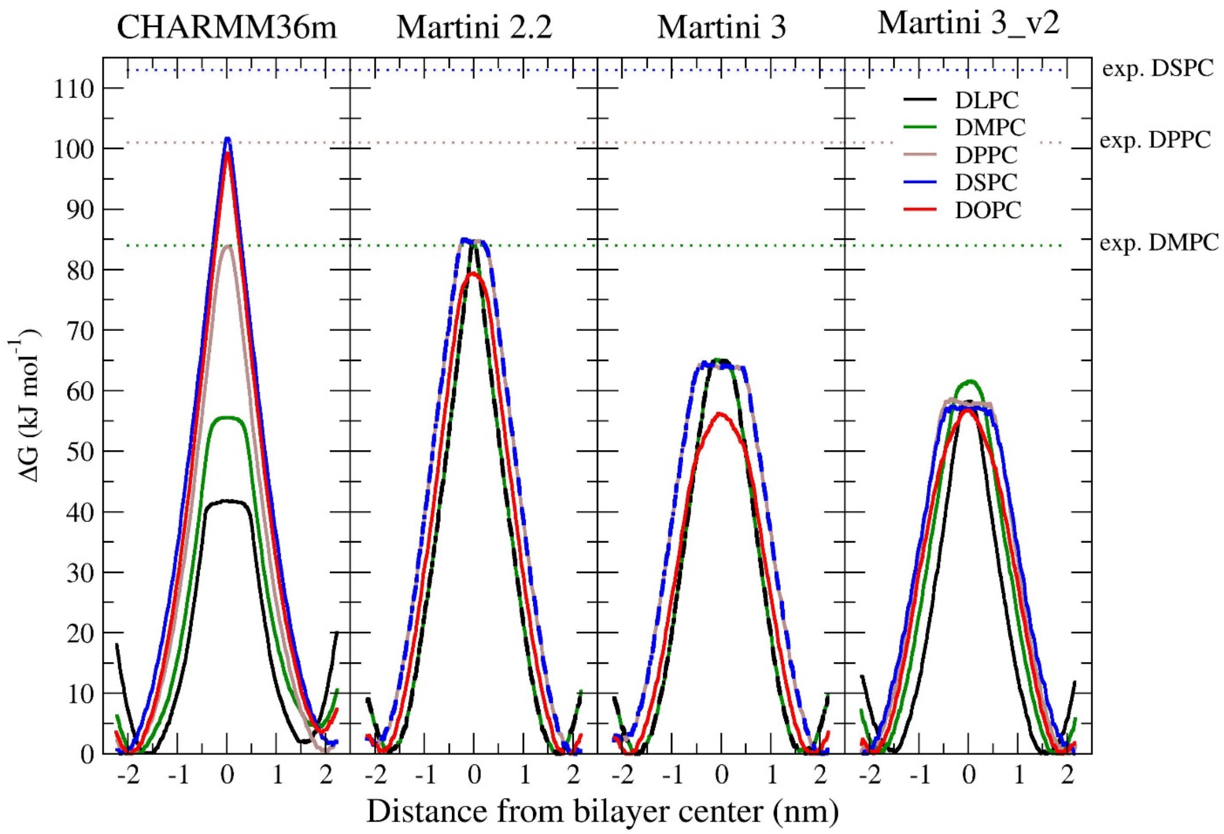

Figure S2. Free energy flip-flop of five phospholipids with varying tail lengths and degrees of saturation, as simulated using the CHARMM36, Martini 2.2, and Martini 3 force fields. From asymmetry, we estimate the error of the profiles to be 6 kJ.mol^{-1}and 3 kJ.mol^{-1} for CHARMM36 and Martini profiles, respectively.

Table S2. Lennard-Jones parameters for all combinations of beads in the studied Martini phospholipids, where Martini 2.2 is used as a reference. New beads in Martini 3 are highlighted in yellow color; increased values are in blue color; decreased values are in red color. For new SN4a and SC1 beads, we use a reference beads of N4a and C1 of the same forcefield, respectively.

Martini 2.2				Martini 3						Martini 3 - refined					
		sig (nm)	eps (kJ.mol^{-1})			sig (nm)	eps (kJ.mol^{-1})	sig (M3 - M2.2)	eps (M3 - M2.2)			sig (nm)	eps (kJ.mol^{-1})	sig (M3-ref. - M3)	eps (M3-ref. - M3)
Q0	Q0	0.470	3.500	Q1	Q1	0.470	3.980	0.00000	0.48000	Q1	Q1	0.470	3.980	0.00000	0.00000
Qa	Qa	0.470	5.000	Q5	Q5	0.470	6.450	0.00000	1.45014	Q5	Q5	0.470	6.450	0.00000	0.00000
Na	Na	0.470	4.000	N4a	N4a	0.470	3.390	0.00000	-0.60979	N4a	N4a	0.470	3.390	0.00000	0.00000
-	-	-	-	SN4a	SN4a	0.410	2.600	-0.06000	-1.39979	SN4a	SN4a	0.410	2.600	0.00000	0.00000
C1	C1	0.470	3.500	C1	C1	0.470	3.390	0.00000	-0.11000	C1	C1	0.470	3.390	0.00000	0.00000
C3	C3	0.470	3.500	C4h	C4h	0.470	3.520	0.00000	0.02000	C4h	C4h	0.470	3.520	0.00000	0.00000
-	-	-	-	-	-			-	-	SC1	SC1	0.410	2.350	-0.06000	-1.04000
Q0	Qa	0.470	4.500	Q1	Q5	0.470	3.790	0.00000	-0.70983	Q1	Q5	0.470	3.790	0.00000	0.00000
Q0	Na	0.470	4.000	Q1	N4a	0.470	4.726	0.00000	0.72621	Q1	N4a	0.470	4.726	0.00000	0.00000
-	-	-	-	Q1	SN4a	0.430	4.236	-0.04000	0.23621	Q1	SN4a	0.430	4.236	0.00000	0.00000
Q0	C1	0.620	2.000	Q1	C1	0.485	2.578	-0.13500	0.57801	Q1	C1	0.485	2.578	0.00000	0.00000
Q0	C3	0.470	2.300	Q1	C4h	0.470	3.776	0.00000	1.47600	Q1	C4h	0.470	3.776	0.00000	0.00000
-	-			-	-			-	-	Q1	SC1	0.443	1.715	-0.04200	-0.86300
Qa	Na	0.470	4.000	Q5	N4a	0.470	4.887	0.00000	0.88700	Q5	N4a	0.470	4.887	0.00000	0.00000
-	-	-	-	Q5	SN4a	0.430	4.386	-0.04000	0.38600	Q5	SN4a	0.430	4.386	0.00000	0.00000
Qa	C1	0.620	2.000	Q5	C1	0.620	2.046	0.00000	0.04600	Q5	C1	0.620	2.046	0.00000	0.00000
Qa	C3	0.470	2.300	Q5	C4h	0.485	2.640	0.01500	0.34000	Q5	C4h	0.485	2.640	0.00000	0.00000
-	-			-	-			-	-	Q5	SC1	0.552	1.066	-0.06800	-0.98000
Na	C1	0.470	2.700	N4a	C1	0.470	2.790	0.00000	0.08986	N4a	C1	0.470	2.790	0.00000	0.00000
-	-	-	-	-	-			-	-	N4a	SC1	0.430	2.160	-0.04000	-0.63000
-	-	-	-	SN4a	N4a	0.430	2.930	-0.04000	-1.06979	SN4a	N4a	0.430	2.930	0.00000	0.00000
-	-	-	-	SN4a	C1	0.430	2.160	-0.04000	-0.54014	SN4a	C1	0.430	2.160	0.00000	0.00000
-	-	-	-	-	-	-	-	-	-	SN4a	SC1	0.410	1.750	-0.02000	-0.41000

Table S3. Parameters of umbrella sampling in coarse-grained (CG) and all-atom (AA) simulations. Bold numbers highlight finer spacing and higher applied force constant. In the AA part, windows where Hamiltonian replica exchange was applied are in bold (16 windows). The gold area in the AA simulations highlights windows generated from up-down pulling, and the blue area highlights windows generated from reversed down-up pulling.

**CG simulations**		**AA simulations**	
**Depth (nm)**	**Force constant ($kJ.mol^{-1}.nm^{-2}$)**	**Depth (nm)**	**Force constant ($kJ.mol^{-1}.nm^{-2}$)**
2,200	3000	2,200	2000
2,090	3000	2,090	2000
1,980	3000	1,980	2000
1,870	3000	1,870	2000
1,760	3000	1,760	2000
1,650	3000	1,650	2000
1,540	3000	1,540	2000
1,430	3000	1,430	2000
1,320	3000	1,320	2000
1,210	3000	1,210	2000
1,100	3000	1,100	2000
0,990	3000	0,990	2000
0,880	3000	0,880	2000
0,770	3000	0,770	2000
0,660	3000	0,660	2000
0,550	3000	0,550	2000
0,440	3000	**0,440**	**3000**
0,330	3000	**0,385**	**3000**
**0,275**	**5000**	**0,330**	**3000**
**0,220**	**5000**	**0,275**	**3000**
**0,165**	**5000**	**0,220**	**3000**
**0,110**	**5000**	**0,165**	**3000**
**0,055**	**5000**	**0,110**	**3000**
**0,000**	**5000**	**0,055**	**3000**
**-0,055**	**5000**	**0,000**	**3000**
**-0,110**	**5000**	**-0,055**	**3000**
**-0,165**	**5000**	**-0,110**	**3000**
**-0,220**	**5000**	**-0,165**	**3000**
**-0,275**	**5000**	**-0,220**	**3000**
-0,330	3000	**-0,275**	**3000**
-0,440	3000	**-0,330**	**3000**
-0,550	3000	**-0,385**	**3000**
-0,660	3000	-0,440	2000
-0,770	3000	-0,550	2000
-0,880	3000	-0,660	2000
-0,990	3000	-0,770	2000
-1,100	3000	-0,880	2000
-1,210	3000	-0,990	2000
-1,320	3000	-1,100	2000

-1,430	3000	-1,210	2000
-1,540	3000	-1,320	2000
-1,650	3000	-1,430	2000
-1,760	3000	-1,540	2000
-1,870	3000	-1,650	2000
-1,980	3000	-1,760	2000
-2,090	3000	-1,870	2000
		-1,980	2000
		-2,090	2000
		-2,200	2000
